# Research on water seepage detection technology of tunnel asphalt pavement based on deep learning and digital image processing

**DOI:** 10.1038/s41598-022-15828-w

**Published:** 2022-07-07

**Authors:** Jiaqi Li, Zhaoyi He, Dongxue Li, Aichen Zheng

**Affiliations:** 1 College of Civil Engineering, Chongqing Jiaotong University, Chongqing, 400074 China; 2 College of Civil Traffic & Transportation, Chongqing Jiaotong University, Chongqing, 400074 China

**Keywords:** Civil engineering, Mathematics and computing

## Abstract

To improve the safety of road tunnel pavement, the research established road tunnel pavement water seepage recognition models based on deep learning technology, and a water seepage area extraction model based on image processing technology to finally achieve accurate detection of water seepage on tunnel pavements. First, the deep learning models EfficientNet water seepage recognition model and MobileNet water seepage recognition model were built, the models were trained with the self-collected pavement seepage data set, and the F1 score was introduced to evaluate the accuracy and comprehensive performance of the two models in predicting different categories of water seepage characteristics. Then three grayscale processing methods, the cvtColor function, mean method and maximum method, six global threshold segmentation methods, Otsu thresholding method, THRESH_BINARY, THRESH_BINARY_INV, THRESH_TRUNC, THRESH_TOZERO and THRESH_TOZERO_INV, three filtering methods, namely Gaussian filtering, median filtering and morphological open operation, as well as small connected domain removal, were used to reduce the noise of the images. Finally, the seepage area image calculation method was proposed based on the processed images to predict the actual pavement seepage area. The results show that the recognition accuracy of the EfficientNet water seepage recognition model is 99.85% and 97.53% in the training and validation sets respectively, which is 2.85% and 0.76% higher than the 97% and 96.77% of the MobileNet model. The average F1 score of the EfficientNet model is 95.22%, which is 5.05% higher than that of the MobileNet model, for the four types of seepage feature images: point seepage, line seepage, surface seepage and no seepage. The cvtColor function for grayscale processing, THRESH_BINARY for threshold segmentation and a combination of median filtering and morphological open operation for image noise reduction can effectively extract the seepage features. The area calculation is performed by the seepage area image calculation method, and the average error between the predicted value and the actual seepage area is 8.30%, which can better achieve the accurate extraction of the seepage area.

## Introduction

With the increase in the number and length of highway tunnels, various tunnel defects have gradually appeared. Water seepage is one of the main factors of tunnel distresses. Water seepage will make the road wet and slippery, causing great hidden dangers to driving safety, and serious damage to the asphalt pavement structure, which will further deteriorate the operating environment and cause traffic safety problems^1–3^. Effective water seepage detection technology plays a vital role in maintaining the asphalt pavement service status of the operating tunnel and ensuring driving safety. Traditional tunnel pavement defect detection mainly relies on manual inspection, which has disadvantages such as low efficiency, high cost, and strong subjectivity in disease evaluation^4,5^. With the rapid development of machine learning and image processing technology, the research proposes to perceive and analyze the water seepage state of the operating tunnel pavement through digital images, which provides a basis for the assessment of the water seepage defect of the tunnel pavement.

Traditional image recognition technology mainly defines the diseased area through shallow features such as image shape, color, texture and gray value, such as the histogram method, edge detection and region growing algorithm. The image is transformed to obtain higher-level feature values for disease identification^6–8^. He et al.^9^ carried out crack image testing on pavement crack images after dimensionality reduction, grayscale correction and filtering, determined the crack interface, and detected asphalt pavement cracks through characteristics such as inclination, Gaussian distribution and edge gradient. Chen et al.^10^ independently constructed a multiscale image analysis method to study pavement diseases. This method can effectively remove the background noise of the picture, sharpen the target edge better, and restrain the boundary movement. Wang et al.^11^ used three-dimensional laser scanning to obtain tunnel seepage disease information, enhanced the edge information of the disease through image binarization, and further analyzed the size of the disease by using a region description algorithm. These methods have a good recognition effect for simple textures, large differences in grayscale, and obvious disease characteristics. However, the effect of image recognition under complex backgrounds is not obvious.

In contrast, deep learning algorithms are completely end-to-end, without human intervention, they can automatically perform abstract expression and analysis based on the characteristics of the original image. It has excellent convenience and operability, and has attracted wide attention from the engineering community^12,13^. Applying deep learning algorithms to the identification and classification of pavement seepage diseases can greatly reduce the feature description, extraction, and recognition based on artificial experience, which is conducive to improving the accuracy and versatility of the seepage recognition algorithm^14,15^. Zhao et al.^16^ constructed a pavement state feature database by segmenting the image, extracting 9-dimensional color feature vectors and 4 texture feature vectors, and proposed a pavement state recognition method based on support vector machine (SVM) to identify wet and slippery states. Zhang et al.^17^ used the SSD-mobilenet architecture to transplant to smart terminals and built a pavement distress database to identify pavement distress images. Wu et al.^18^ combined DenseNet and deconvolutional network to form an end-to-end multiscale fully convolutional neural network, which segmented and identified cracks and defects in the complex fine-grained background of asphalt pavement.

CNNs have become the most representative neural network in the field of deep learning. It can work with complex environmental information, vague background knowledge, and unclear reasoning rules^19–21^. The traditional water seepage disease recognition algorithm uses a large number of image processing processes, which takes a long time and the recognition rate and accuracy are not high^22^. However, using the CNN model does not require a large number of image processing procedures. The image can be directly used as input for training. The model can automatically learn the pixel difference of the water seepage image to quickly and accurately complete the classification target^23–25^.

In this work, the convolutional neural network model based on deep learning is designed according to the classical network structure. The images of tunnel pavement seepage are collected, and the accuracy of the models is tested by establishing a data set and a verification set. To further improve the accuracy of recognition, the extended network EfficientNet recognition model based on the CNN backbone network is used to achieve the goal of correctly identifying pavement water seepage. On the basis of identifying pavement water seepage, the geometric characteristics of pavement water seepage are extracted based on semantic segmentation, and the area of water seepage is calculated, which lays the foundation for maintenance evaluation.

## EfficientNet model

**Figure 1:**
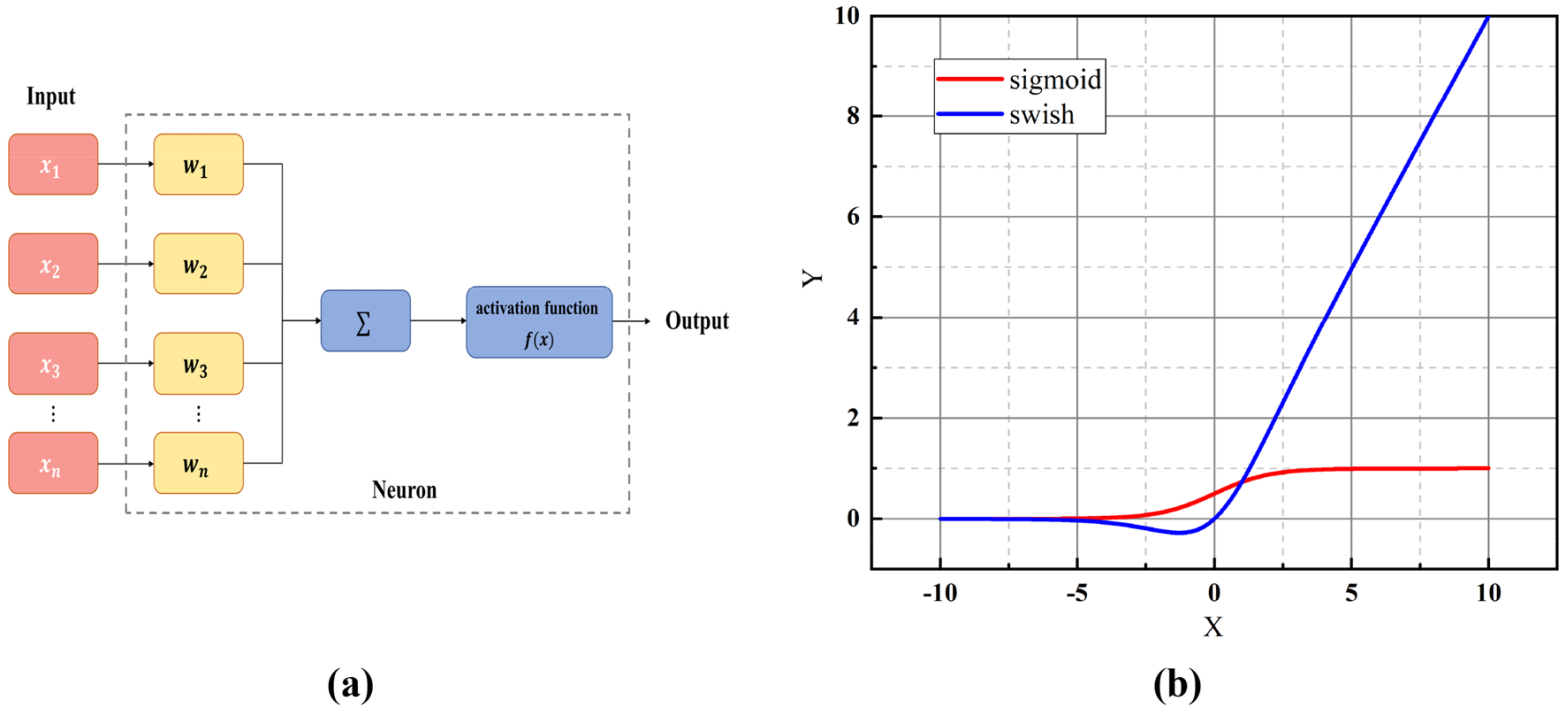
Activation function (**a**) Activation function flowchart; (**b**) Sigmoid activation function and Swish activation function.

The EfficientNet model is a set of backbone feature extraction networks based on CNN proposed by Tan M et al.^26^ in 2019. CNN models are usually trained under known hardware resource conditions, while EfficientNet is a model generated by the MnasNet model implemented by a reinforcement learning algorithm. The three dimensions of the model's depth, width (the number of channels in the feature map), and resolution (the input image size) are simultaneously scaled and finally expanded to form the EfficientNet series network models^27^. As shown in Fig. 1, the EfficientNet network model realizes the comprehensive consideration of the three factors of depth, width, and resolution. Compared with other network models, the EfficientNet series network models can maintain a high classification accuracy rate with a small number of model parameters^28,29^.

### The structure of the EfficientNet model

The EfficientNet model consists of four parts: mobile inverted bottleneck convolution (MBConv) module, convolution layer, pooling layer, and fully connected layer. MBConv is obtained through neural network architecture search, and the module structure is similar to depthwise separable convolution. It gives the model a random depth, shortens the practice required for model training, and improves the performance of the model. The function of the convolution layer is to extract different features of the input picture and hand them to the subsequent high-level neurons for processing. The first layer of the convolution layer can only extract some low-level local features, such as edges, lines, and corners, and more complex features can be iteratively extracted from low-level features through more layers of convolution. Usually after the convolution layer, features with large dimensions are obtained. The role of the pooling layer is to divide the features into several regions and remove the maximum or average value to obtain new features with smaller dimensions while reducing the risk of overfitting. The one used in this paper is global average pooling. The fully connected layer combines all the local features into a global feature, that is, fully connects the results after convolution and pooling, and turns multidimensional vectors into one-dimensional vectors.

### Activation function

The main role of the activation function is to introduce nonlinearity into the network. If there is no activation function, the output signal will be a simple linear function with limited complexity. The mapping ability of learning complex functions from data is smaller, and the ability to extract features is weakened. Commonly used activation functions include the sigmoid function, Tanh function and ReLU function. The working principle of the activation function is shown in Fig. 1a. The input layer input ${x}_{i}$ is multiplied by the weight ${w}_{k}$ and then accumulated, and the activation $y$ is obtained through the activation function.

Compared with the underfitting problem when the input value of the tanh function is too large or too small, the ReLU function has the limitation of overfitting and forced sparse processing to cause neuron necrosis. In this paper, the Swish activation function is used. The Swish activation function is a variant of the sigmoid activation function. The expression of the sigmoid function is as follows:

1 $$ Sigmoid\left( x \right) = \frac{1}{{1 + e^{ - x} }} $$

The expression of the Swish function is as follows, where β is an adjustable parameter:

2 $$ Swish\left( x \right) = x \cdot Sigmoid\left( {\beta x} \right) $$

The Swish function has the characteristics of no upper bound, lower bound, smooth curve, and non-monotonic function. The performance of the Swish function in shallow networks is not particularly prominent, but as the depth of the network increases, the performance of the Swish function becomes increasingly prominent. The EfficientNet network selected in this paper has a more complex model and a deeper network structure, so it is more suitable to use the Swish function as the activation function.

### Feature convolution module

The mobile flip bottleneck convolution first performs 1*1 point-by-point convolution on the input feature map, changes the output channel dimension according to the expansion ratio, and then performs batch normalization (BN) and an activation function (Swish). If the expansion ratio is 1, then the point-by-point convolution, BN and activation functions are directly skipped. Then, we perform k*k depthwise convolutions, finally introduce compression and excitation operations, and finally end with a 1*1 point-by-point convolution to restore the original channel dimension.

The SENet module in the MBConv module is a feature map operation based on attention. First, the SENet module compresses the feature map, performs global average pooling in the channel dimension direction, and obtains the global feature map in the channel dimension direction. Then the global feature is excited, and the 1*1 convolution equal to the number of global feature dimensions C is multiplied by the activation ratio R to perform convolution on the feature map. The relationship between each channel is learned, and then the weights of different channels are obtained through the sigmoid activation function. Finally, the original feature map is multiplied to obtain the final feature.

To solve the gradient disappearance and gradient explosion caused by the excessive number of model convolution layers. The MBConv module introduces the short-circuit path of the residual module. This makes the gradient spread coherently in the very deep network to prevent the gradient from superimposing.

### Loss function

The loss function is used to estimate the degree of inconsistency between the predicted value and the true value of the model built^30^. And the loss function used for the seepage recognition model established in this paper is the binary_crossentropy function, whose expression is shown below.

3 $$ Loss = - \frac{1}{N}\mathop \sum \limits_{1}^{N} y_{i} \cdot \log \left( {p\left( {y_{i} } \right)} \right) + \left( {1 - y_{i} } \right) \cdot \log \left( {1 - p\left( {y_{i} } \right)} \right) $$

In the formula, $N$ is the output size, $i$ is the category, ${y}_{i}$ is the label, and $p({y}_{i})$ is the probability that the output is labeled with ${y}_{i}$, where each $i$ in $i\in [1,N]$ is independent of each other and does not interfere with each other, so it is suitable for multilabel classification.

## Design of the water seepage recognition model

To establish a deep learning model that can classify water seepage images of asphalt pavement and extract and calculate water seepage characteristics, this paper uses two models, the MobileNet model and the EfficientNet model, to learn the water seepage characteristics of asphalt pavement. The models are designed based on the Python language and Keras development framework.

### Data collection and processing

**Figure 2:**
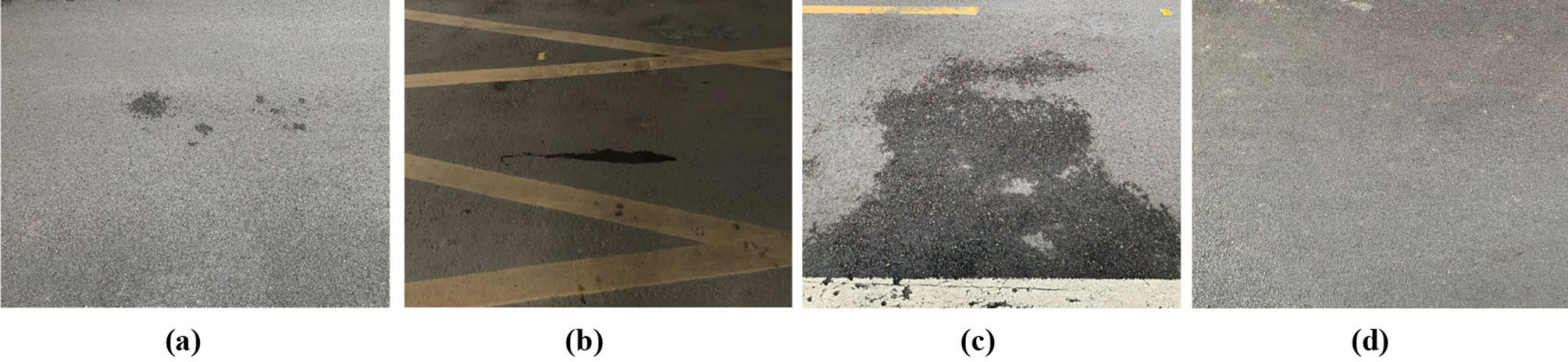
Classification of different water seepage characteristics (**a**) point seepage; (**b**) line seepage; (**c**) surface seepage; (**d**) no seepage.

The models designed in this paper are mainly used to classify and identify the four types of water seepage characteristics of tunnel asphalt pavement: point seepage, line seepage, surface seepage, and no seepage, as shown in Fig. 2. Using the mobile phone, 200 pictures of each of the 4 types of water seepage characteristics of asphalt pavement in different tunnels were collected. The four types of features are labeled A, B, C, and D, and they are stored in different folders. Since deep learning model training requires a large amount of data, and considers the time cost of collecting data in real scenes and the influence of various environmental factors, to ensure the diversity of data, the collected data are expanded and enhanced.

As shown in Table 1, the data set is expanded by means of flipping up and down, mirroring, changing the brightness, and Gaussian blurring. The four types of pavement water seepage characteristic images were increased to 600, which basically met the requirements of model training. Not only can it effectively prevent the model from overfitting, but it can also improve the model's recognition rate of pavement seepage.

**Table 1:**
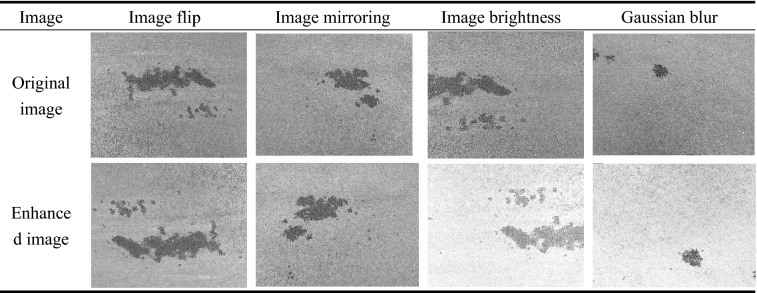
Data set expansion.

### MobileNet model construction

MobileNet is a typical representative of lightweight networks. It introduces deep separable convolution based on the classic CNN model, and replaces the pooling layer and part of the fully connected layer with convolution layers^31,32^. Standard convolution combines a set of convolution kernels and inputs data into a single-channel feature output. The convolution form of depth separable convolution is factorization. The standard convolution is solved into two parts: deep convolution and pointwise convolution. A single convolution kernel of a fixed size is applied to each input channel through deep convolution, and then channel information is fused and output through point-by-point convolution.

The MobileNet model established in this paper is first a 3*3 standard convolution. Then pointwise convolution is used alternately with the depthwise convolution layer with a step length of 1 or 2. Then , average pooling is used to change the characteristic value into 1*1, and a fully connected layer is added according to the prediction of four types of water seepage. Finally, there is a softmax layer. There are 28 layers of depthwise convolution and pointwise convolution in this network model. The network structure is shown in Table 2.

**Table 2:** MobileNet network structure.

	Type	Stride	Filter shape
	Conv	s2	3 × 3 × 3 × 32
	Conv dw	s1	3 × 3 × 32
	Conv	s1	1 × 1 × 32 × 64
	Conv dw	s2	3 × 3 × 64
	Conv	s1	1 × 1 × 64 × 128
	Conv dw	s1	3 × 3 × 128
	Conv	s1	1 × 1 × 128 × 128
	Conv dw	s2	3 × 3 × 128
	Conv	s1	1 × 1 × 128 × 256
	Conv dw	s1	3 × 3 × 256
	Conv	s1	1 × 1 × 256 × 256
	Conv dw	s2	3 × 3 × 256
	Conv	s1	1 × 1 × 256 × 512
5 ×	Conv dw Conv	s1 s1	3 × 3 × 512 1 × 1 × 512 × 512
	Conv dw	s2	3 × 3 × 512
	Conv	s1	1 × 1 × 512 × 1024
	Conv dw	s2	3 × 3 × 1024
	Conv	s1	1 × 1 × 1024 × 1024
	Avg Pool	s1	Pool 7 × 7
	FC	s1	1024 × 1000
	Softmax	s1	Classifier

### EfficientNet model construction

**Figure 3:**
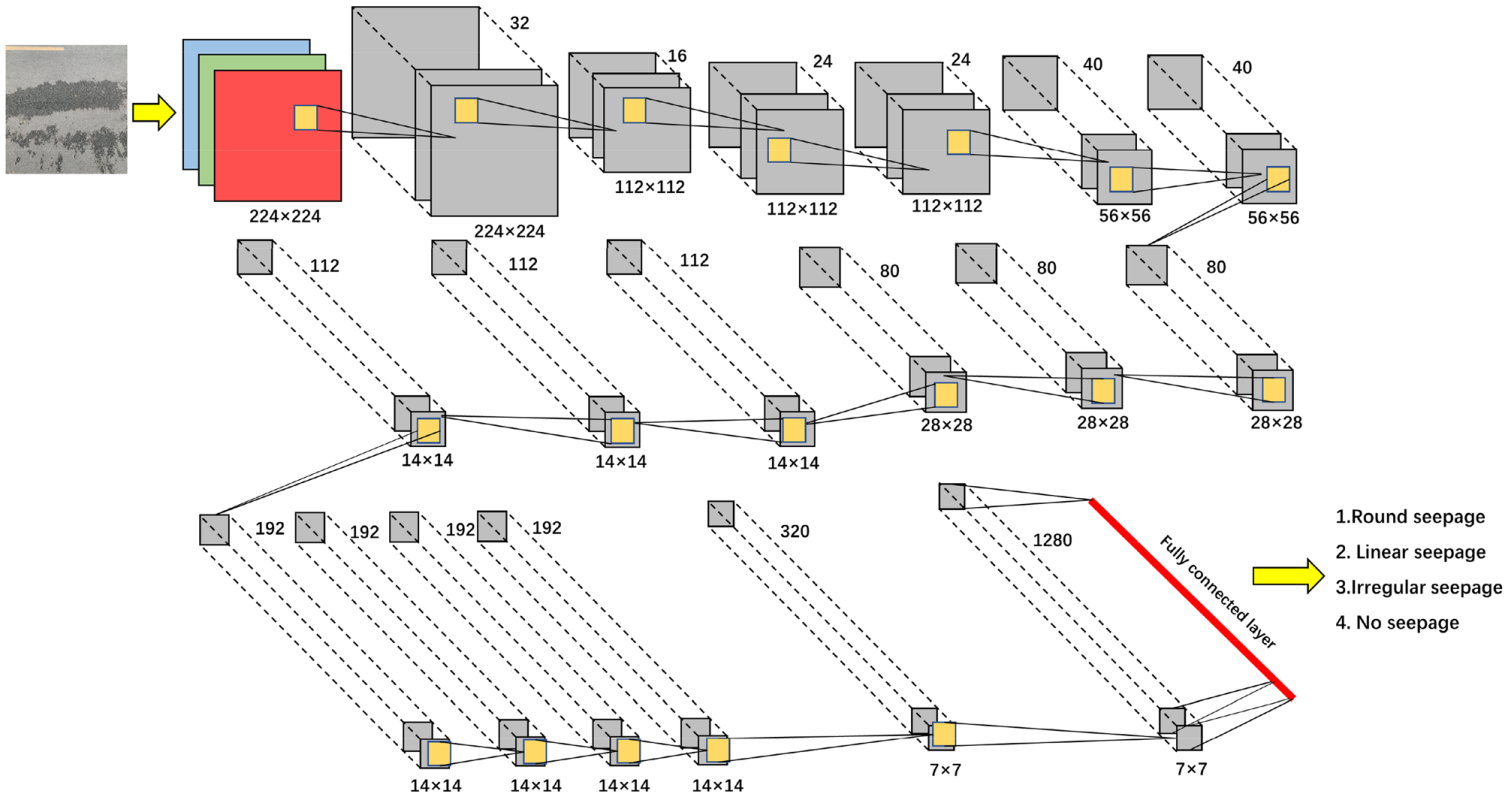
EfficientNet network structure.

The EfficientNet model established in this paper is divided into 9 parts, for a total of 18 layers of neural networks. The first part is an ordinary convolutional layer with a convolution kernel of 3*3 and a step size of 2, which contains BN and Swish. The second part is the MBConv structures with the 3*3 convolution kernel. Parts 3 ~ 8 are the MBConv structures that expand the channel of the input feature matrix to 6 times the original, and the convolution kernels are 3*3, 5*5, 3*3, 5*5, 5*5, and 3*3, respectively. The 9th part consists of a 1 × 1 convolutional layer (including BN and Swish), an average pooling layer and a fully connected layer. The network structure is shown in Fig. 3.

**Figure 4:**
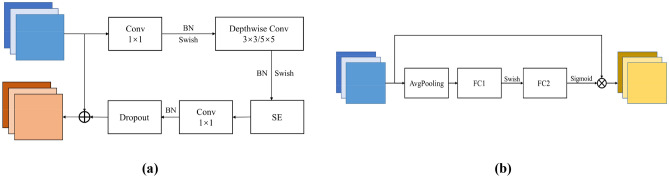
MBConv module and SE module (**a**) MBConv module; (**b**) SE module.

As shown in Fig. 4, the MBConv structure is mainly composed of a 1*1 ordinary convolution for dimension upgrade, a deep convolution with a convolution kernel size of 3*3 or 5*5, an SE module, and a 1*1 ordinary convolution for dimensionality reduction and a dropout layer. In the SE module, the number of nodes in the first fully connected layer is 1/4 of the channels input to the MBConv feature matrix, and the Swish activation function is used. The number of nodes in the second fully connected layer is the same as the number of feature matrix channels output by the deep convolutional layer, and the sigmoid activation function is used.

### Comparison of model results

The test environment is as follows: Windows10 operating system, Intel i7-4720HQ processor, NVIDIA GeForce RTX2060, Python 3.8, Keras 2.2.5, and TensorFlow 1.14.0. The 2400 images after image enhancement are used as the training data set, 70% of which are used as the training set, 20% are used as the test set, and 10% are used as the validation set for testing. To ensure that the two models are performed in the same environment, the input image size is 224*224*3, the training time epoch is 60, and the loss function is binary_crossentropy. The hyperparameters used in this experiment are set as follows: the batch size is 32, the optimizer uses Adam, and the initial learning rate is 0.001.

**Figure 5:**
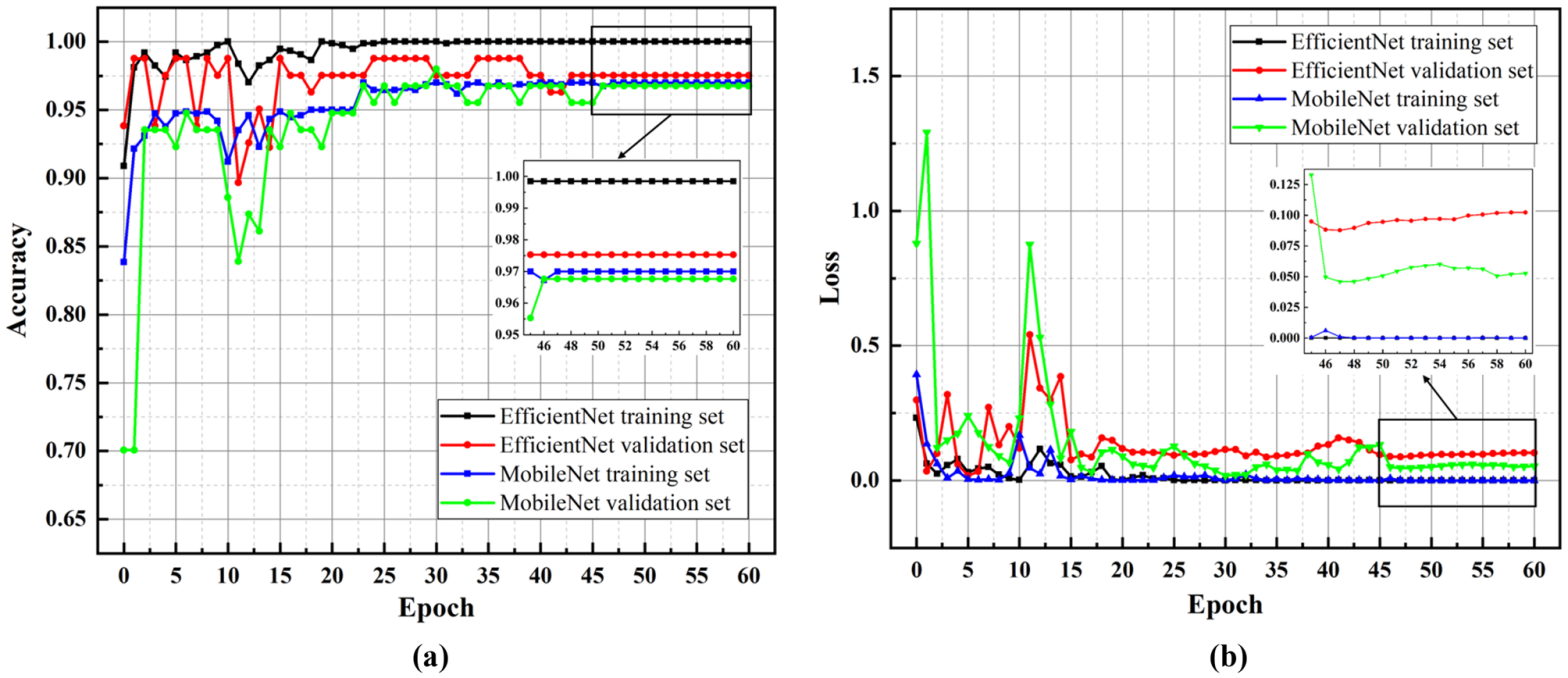
The MobileNet model and the EfficientNet model training results (**a**) Line graph of the accuracy of the training set and validation set with the epoch of training; (**b**) Line graph of the loss value of the training set and the validation set with the epoch of training.

The training results of the MobileNet and EfficientNet models are shown in Fig. 5.

As shown in Fig. 5, the accuracy of the first epoch training set is 83.84%, and the loss value is 0.3929 using the MobileNet model for water seepage recognition. After 60 epochs of training, the accuracy of the model in the training set is 97%, and the loss value is 3.4 × 10^−4^. The accuracy rate of the validation set increased from 70.07 to 96.77%, an increase of 26.7%, and the loss value was reduced from 0.8786 to 0.0527, a decrease of 0.8259. In the whole training process, the accuracy rate increases with increasing training, and the loss decreases with increasing training, which meets the training requirements as a whole. However, the fluctuations in the accuracy and loss of the verification set are significantly larger than those of the training set. This is because the amount of data in the verification set is less than that of the training set, and does not affect the judgment of the result.

After 60 epochs of training, the accuracy of the EfficientNet model training set increased from 90.88 to 99.85%, and the loss was reduced from 0.2318 to 1.67 × 10^−5^. The accuracy of the validation set has increased from 93.83% to 97.53%, an increase of 3.7%, and the loss was reduced from 0.2976 to 0.1023, which is a decrease of 0.1973. Compared with the MobileNet model, the EfficientNet model validation set has a smaller range of accuracy and loss, and the model has a better recognition effect for images.

To further compare the accuracy of the two models for the prediction of water seepage feature images, the F1 Score index is introduced for evaluation. The F1 score takes into account the precision and recall of the classification model, and is the harmonic average of the accuracy and recall of the model. The equation is as follows:

4 $$ Pr = \frac{TP}{{TP + FP}} $$

In Eq. (4): Pr is the accuracy rate; TP is the true positive prediction; FP is the false positive prediction.

5 $$ Re = \frac{TP}{{TP + FN}} $$

In Eq. (5): Re is the recall rate; FN is the false negative prediction.

6 $$ F1 Score = \frac{2 \cdot Pr \cdot Re}{{Pr + Re}} $$

The 480 test set images used in this experiment were predicted, including 102 for point seepage, 121 for line seepage, 139 for surface seepage, and 118 for no seepage, and the predicted results were evaluated for each category. A weighted average was used to obtain the average index of the model. The results are shown in Table 3.

**Table 3:** Statistics table of water seepage prediction results.

Model	Characteristic type	TP	FP	FN	TN	Pr	Re	F1 score
Mobilenet	Point seepage	90	14	12	376	0.8654	0.8824	0.8738
	Line seepage	105	14	16	361	0.8824	0.8678	0.8750
	Surface seepage	120	16	19	344	0.8824	0.8633	0.8727
	No seepage	118	3	0	359	0.9752	1.0000	0.9874
	Average value					0.9016	0.9021	0.9017
EfficientNet	Point seepage	96	10	6	374	0.9057	0.9412	0.9231
	Line seepage	116	8	5	356	0.9355	0.9587	0.9469
	Surface seepage	128	5	11	347	0.9624	0.9209	0.9412
	No seepage	117	0	1	363	1.0000	0.9915	0.9957
	Average value					0.9528	0.9521	0.9522

As shown in Table 3, the EfficientNet model predicts 90.57% accuracy for point seepage, 93.55% accuracy for line seepage, 96.24% accuracy for face seepage, and 100.00% accuracy for no seepage. The average prediction accuracy of the EfficientNet model for the four types of seepage features after training was 95.28%. The model has the highest accuracy for no seepage among the four types of features. Since the grayscale distribution of the no-seepage images is uniform and the difference in the grayscale value of each pixel is small, the model can better distinguish from the images with seepage that have a large difference in grayscale when training the features. The accuracy of the EfficientNet model is higher than that of the point seepage and line seepage among the three types of seepage, indicating that the model has the best learning ability for surface seepage features, and the accuracy of point seepage prediction is relatively weak, probably because the features of point seepage and surface seepage are similar. This may be due to the similarity of the characteristics of point seepage and surface seepage in that the model misidentified some point seepage pictures as surface seepage.

The average prediction accuracy of the MobileNet model for the four types of seepage features is 90.16%, from highest to lowest: no seepage, line seepage, surface seepage and point seepage. In the prediction of no seepage, three point seepage images were mistaken as no seepage, which indicates that the model is weaker than the EfficientNet model in the extraction of seepage features. In the prediction of the four types of seepage features, the prediction accuracies of the EfficientNet model are higher than those of the MobileNet model, and the average prediction accuracy of the EfficientNet model is 5.12% higher than that of the MobileNet model. The EfficientNet model is better than the MobileNet model in terms of prediction accuracy for water seepage images.

The average F1 scores of the EfficientNet model and MobileNet model are 95.22% and 90.17% respectively, both of which reach above 90%, indicating that both models can perform the identification of pavement water seepage features well. The EfficientNet model is not only better than the MobileNet model in terms of accuracy, but also has higher recall and F1 score than MobileNet, and the average F1 score is 5.05% higher than MobileNet. In summary, the EfficientNet model is more accurate and has better performance for pavement water seepage recognition.

## Geometric characteristics of pavement water seepage

After the pavement water seepage images are classified, further information such as the shape and area of the road water seepage need to be obtained before the driving safety and the degree of pavement damage can be evaluated. The collected pavement water seepage set contains most of the pavement information, and image processing technologies need to be used to effectively extract the image water seepage information to reduce the interference caused by other information and provide data for road condition evaluation.

### Image processing

To ensure that high quality water seepage characteristic information can be obtained, reduce the loss of water seepage information caused during image processing, and improve the accuracy of water seepage geometric feature extraction, the resolution of the picture is uniformly adjusted to 3500 × 3500 before image processing.

#### Gray processing

Since the collected pavement water seepage images are all color digital images, the color of each pixel depends on the color components R, G, and B, each component has 255 values that can be taken, and the color range of a pixel point is 255*255*255. After the color images are grayscale scaled, the R, G, and B components of each pixel are the same, and each pixel has only 255 color ranges, which greatly reduces the amount of image calculation and recognition time. Similar to color images, grayscale images also reflect the overall image, the distribution and characteristics of the layout chromaticity and grayscale levels.

This paper uses three gray processing methods, namely, cvtColor, meanGray and maxGray.

**Figure 6:**
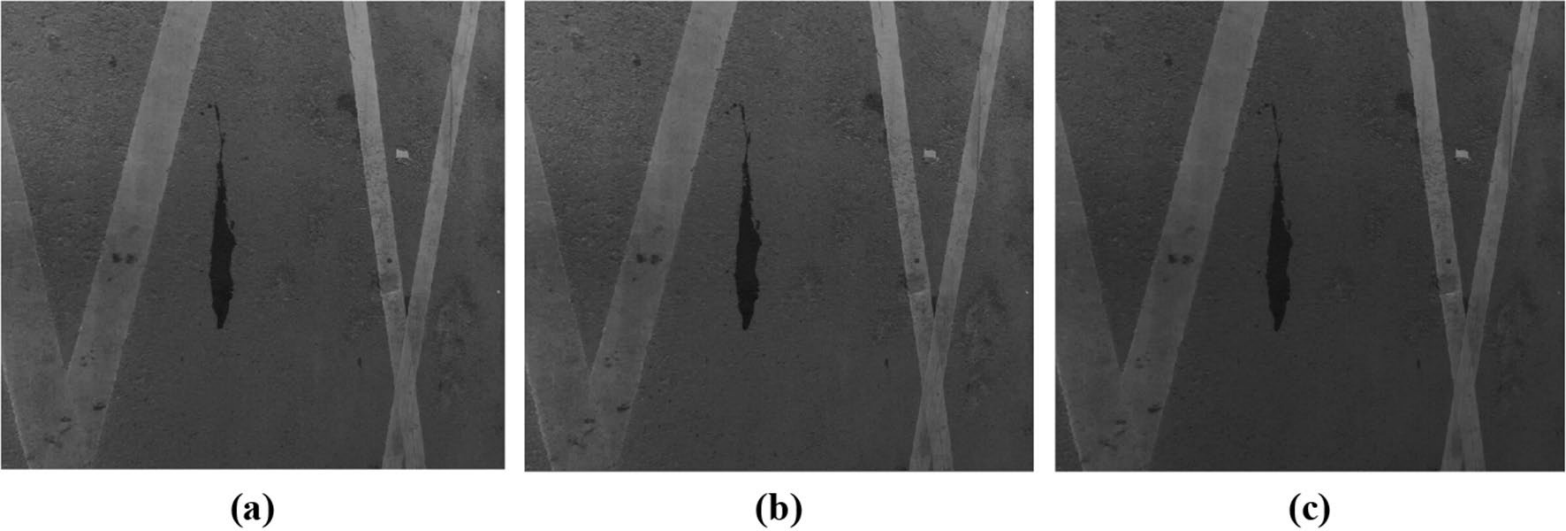
Image gray processing (**a**) cvtColor; (**b**) meanGray; (**c**) maxGray.

It can be seen from Fig. 6 that after using maxGray, the overall image is dark. Although it is beneficial to more completely extract water seepage characteristics, it will cause considerable unnecessary noise when thresholding the image, which increases the difficulty of image processing. After the meanGray, the edge pixels of the water seepage characteristics are similar, and the edge contour of the water seepage characteristics cannot be well recognized. After using the cvtColor, the edges contour of the water seepage characteristics are obvious, and the feature pixels of high and low brightness are better improved. Compared with the other two methods, the water seepage characteristics are more obvious. Therefore, cvtColor is selected as the method for gray processing.

#### Threshold segmentation

Threshold segmentation is binarization. The target feature is separated from the image background according to a certain threshold, and the image can be segmented by using the gray difference between the target feature area and the background area. The image after binarization has only two colors of black and white, that is, the gray value is only 0 and 255, which can better highlight the characteristic area of water seepage.

The six threshold segmentation methods used in this article are: Between-cluster variance method (OTSU), THRESH_BINARY, THRESH_BINARY_INV, THRESH_TRUNC, THRESH_TOZERO, THRESH_TOZERO_INV.

**Figure 7:**
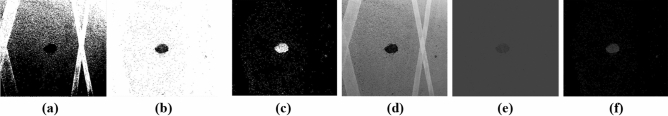
Threshold segmentation (**a**) OTSU; (**b**) THRESH_BINARY; (**c**) THRESH_BINARY_INV; (**d**) THRESH_TRUNC; (**e**) THRESH_TOZERO; (**f**) THRESH_TOZERO_INV.

According to many tests, when the threshold is set to 65, the water seepage characteristics are displayed intact, and the image is in the best state. The image processed by the above method is shown in Fig. 7.

It can be seen from Fig. 7 that since the asphalt pavement and water seepage characteristics are all black to different degrees under natural conditions, after gray processing, similar gray levels will be obtained. Therefore, when the OTSU is used, the foreground and the background cannot be distinguished more accurately. A certain gray level is also retained in THRESH_TRUNC. Although the water seepage characteristic area is more prominent than the gray processing, the background is not completely eliminated, which is not conducive to the subsequent extraction of geometric features. Although THRESH_BINARY will produce some small connected domain noise, it shows relatively obvious water seepage characteristics compared with THRESH_TOZERO and THRESH_TOZERO_INV. Therefore, the THRESH_BINARY method is selected as the threshold segmentation method of water seepage images.

#### Image denoising

Image noise is a random change in image color, which is unavoidable during image shooting and transmission, and is redundant interference information. In asphalt pavement, because the asphalt, aggregate and water seepage colors are similar, considerable noise will be generated. These noises do not belong to the scope of water seepage, but they will be included in the scope of the water seepage area when calculating the geometric characteristics of water seepage. To eliminate this effect, the image needs to be denoised. After a large number of experiments, it is found that the effect of using a single method to reduce noise is not very obvious. This paper uses Gaussian filtering combined with morphological open operation and median filter combined with morphological open operation to reduce noise.

**Figure 8:**
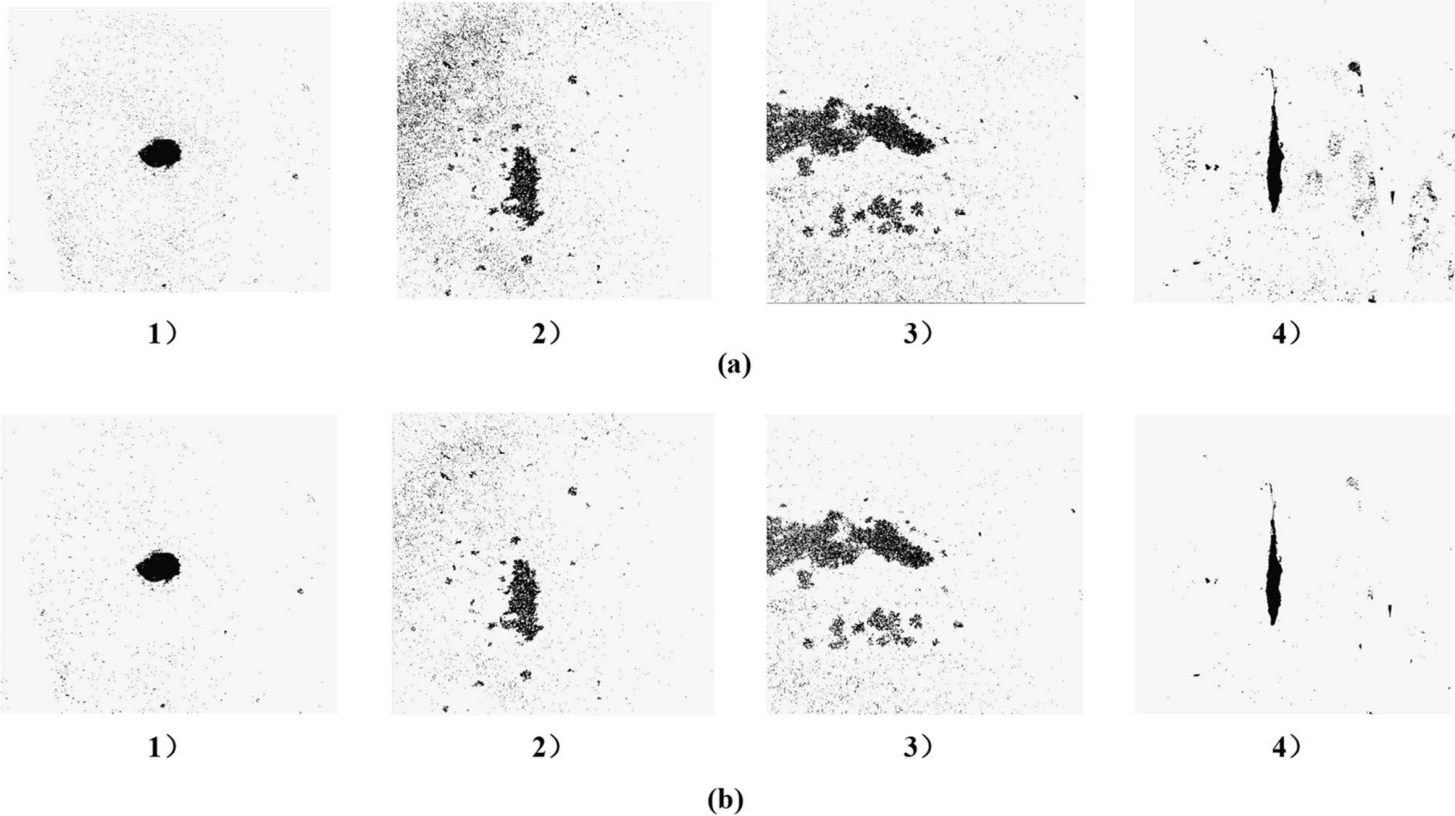
Image denoising (**a**) Gaussian filtering combined with morphological open operation: 1) point seepage; 2) surface seepage; 3 surface seepage; 4) line seepage; (**b**) median filter combined with morphological open operation: 1) point seepage; 2) surface seepage; 3) surface seepage; 4) line seepage.

It can be seen from Fig. 8 that the noise reduction effect of the noise reduction method combining Gaussian filtering with a kernel of 5*5 and morphological open operation is not very obvious, and the image still has considerable noise, which cannot highlight the water seepage characteristics. Compared with the former, the method of the median filter combined with morphological open operation can reduce most of the noise in the images, has a better filtering effect on salt and pepper noise, and has less loss of water seepage characteristics. Therefore, the method of the median filter combined with the morphological open operation is used to reduce the noise of the image.

#### Removal of small connected domains

**Figure 9:**
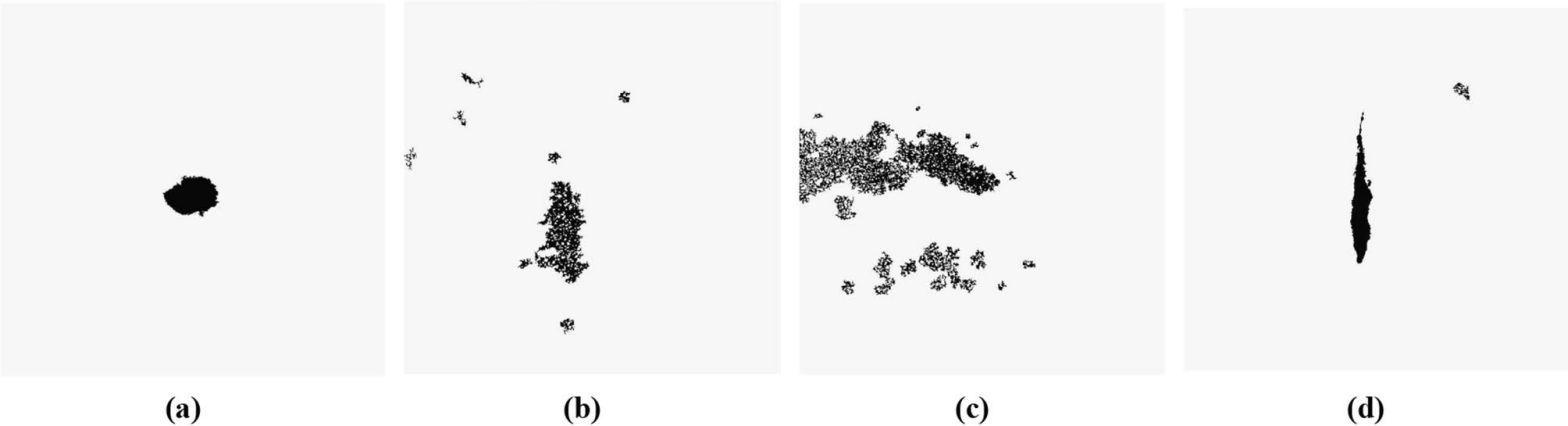
Water seepage feature images after removing small connected domains (**a**) point seepage; (**b**) surface seepage; (**c**) surface seepage; (**d**) line seepage.

After the image noise is reduced, there are still many small black pixels that are not characteristic of water seepage. These small connected domains composed of adjacent pixels with the same pixel level have a great impact on the accumulation of subsequent water seepage characteristic pixels. The definition of connected domains is generally divided into two types: 4 domains and 8 domains. To reduce the loss of edge pixels of the water seepage feature, the connected domains of the no-seepage characteristic are removed. The black connected domain around the seepage feature is removed by the 4 domains method. For the pixels in the water seepage characteristic that were lost in the previous image processing, an 8 domains method is used to fill in. This makes the water seepage characteristic pixels more complete and closer to the actual water seepage area. The water seepage characteristics removed by the small connected domains are shown in Fig. 9.

### Extraction of pavement water seepage geometric features

The processed images only have water seepage characteristics. In the image, pixels with a level of 0 are all water seepage characteristics. Through statistics and analysis of pixels with a level of 0, a method for extracting geometric information of pavement water seepage is proposed. A coordinate system (x, y, z) is established based on the space where the actual pavement is located, and the pavement level coincides with the xoz plane of the coordinate system. The imaging plane coordinate system (u, v) is established for the plane where the camera is located, and the uv plane is parallel to the xoz plane. Through the measurement of the actual pavement, the length and width information of the xoz plane collected in the image is obtained, and a proportional relationship is established between the length and width pixels of the uv plane. Through scale conversion, the actual pavement length represented by each pixel and the actual road pavement information represented by one pixel can be accurately obtained. Through the accumulation of pixels, the actual pavement water seepage area and other information can be obtained.

**Figure 10:**
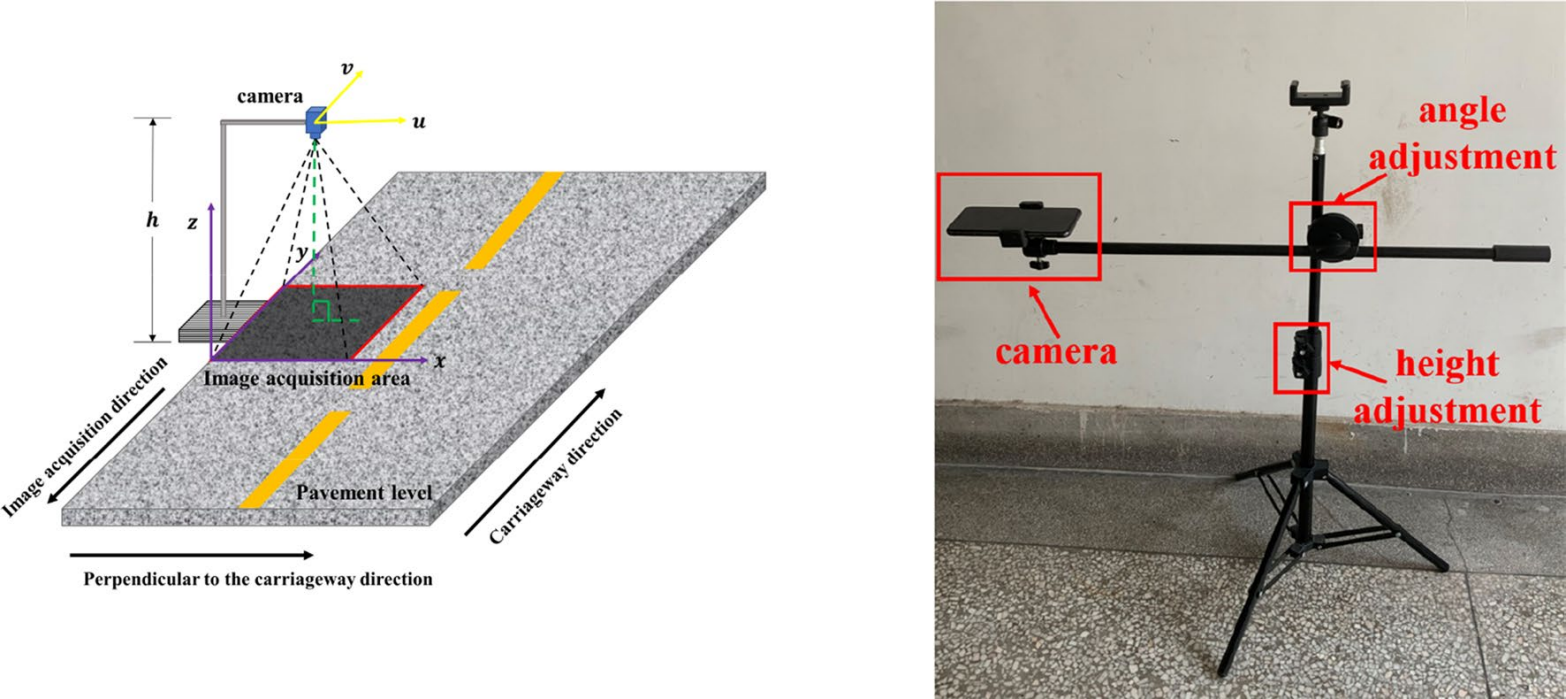
Field collection device.

As shown in Fig. 10, to avoid the interference of the shooting angle and shooting distance on the accuracy of water seepage feature recognition, it is necessary to fix the camera parameters and the height and angle of the device. In this experiment, the height of the camera from the ground is fixed at 150 cm, the optical axis of the camera is perpendicular to the pavement level, the aspect ratio of the photo is 1:1, and the image pixels are 3500 × 3500.

From Table 4, the average error of point seepage is 7.40%, the average error of line seepage is 8.05%, and the average error of surface seepage is 9.33%. The effects of this method in the extraction of point seepage areas and line seepage areas are better than those of surface seepage areas. It may be that the water seepages at the edges of the surface seepage are filtered when the images are filtered, which makes the error between the predicted water seepage area and the actual water seepage area larger. The average error of the three types of water seepage area extraction is 8.30%, which is controlled within 10%. This result shows that this method has a good effect on the extraction of water seepage geometric features.

**Table 4:** Area calculation of the water seepage area.

No	Type	Number of pixels	Predicted value	Error	Original picture of pavement water seepage	Characteristic extraction result
1	Point seepage	121,116	320.34 cm^2^	24.86 cm^2^		
2		143,366	379.19 cm^2^	26.31 cm^2^		
3		65,349	172.84 cm^2^	13.86 cm^2^		
4		95,295	252.05 cm^2^	23.45 cm^2^		
5	Line seepage	141,274	373.66 cm^2^	32.04 cm^2^		
6		88,610	234.36 cm^2^	20.94 cm^2^		
7		1,049,508	2775.84 cm^2^	291.36 m^2^		
8	Surface seepage	230,635	610.01cm^2^	58.89 cm^2^		
9		557,642	1474.91 cm^2^	160.29 cm^2^		
10		812,174	2148.12 cm^2^	217.68 cm^2^		

## Limitations and future prospects

In the current research, water seepage detection on tunnel pavement is first recognized using the EfficientNet and MobileNet models; then, the images are noise reduced using different image noise reduction techniques, and finally, the actual pavement seepage areas are predicted from the images using coordinate matrix transformation. Water seepage recognition is only classified without locating the specific location of the seepage in the images, and the methods used in image noise reduction and seepage area segmentation require manual parameter adjustment for large batch image processing, which takes considerable time.

In subsequent research, to better identify the seepage geometry and develop corresponding maintenance measures for different forms of seepage, it is necessary to combine deep learning target detection with instance segmentation. Target detection will be used to achieve multiobjective detection of seepage diseases, and instance segmentation will be used to solve the problem of accurate extraction of seepage disease features in large batches, so as to finally achieve accurate and efficient detection of multiple disease features in images. The pixel-level separation of seepage geometry using images will be the focus and difficulty in future work.

## Conclusion

1. For the digital image recognition problem of tunnel pavement water seepage, two deep learning water seepage recognition models, EfficientNet and MobileNet, are established, and water seepage category prediction is performed by training and analyzing 1920 images and 480 images. The results show that the training accuracy of the EfficientNet model for pavement seepage recognition is 99.85% and the average prediction accuracy is 95.22%, which are 2.85% and 5.05% higher than the training accuracy of 97.00% and prediction accuracy of 90.17% of the MobileNet model, respectively, indicating that the EfficientNet model can better identify pavement water seepage.
2. For the pavement seepage image noise reduction problem, three methods, the cvtColor, mean method and maximum method are used for grayscale processing; six methods, Otsu thresholding, THRESH_BINARY, THRESH_BINARY_INV, THRESH_TRUNC, THRESH_TOZERO and THRESH_TOZERO_INV, are used for global threshold segmentation; and three methods, Gaussian filtering, median filtering and morphological open operation, are used for image filtering. The results show that the use of cvtColor can enhance the edges of seepage feature contours, the use of the THRESH_BINARY method can better threshold the image segmentation, the use of median filtering and morphological open operation can better reduce the image pepper noise, and finally the use of a small connected domain can remove excess noise and highlight the seepage disease details. Combining these methods for image processing can improve the overall image quality and be more conducive to disease feature extraction.
3. For the extraction of tunnel pavement seepage features, the coordinate matrix relationship transformation is used to correlate the image pixel points with the actual pavement length and width information, and the actual pavement water seepage area can be predicted through the calculation of water seepage pixel points. The experimental results show that the method has the best prediction effect on point water seepage with an average error of 7.40% and the least prediction effect on face water seepage among the four kinds of defects with an average error of 9.33%, but the total error for the four kinds of defects is controlled within 10%, which can better achieve the accurate analysis of the water seepage area.
